# Maternal challenges of exclusive breastfeeding and complementary feeding in Ghana

**DOI:** 10.1371/journal.pone.0215285

**Published:** 2019-05-02

**Authors:** Anthony Mwinilanaa Tampah-Naah, Akwasi Kumi-Kyereme, Joshua Amo-Adjei

**Affiliations:** 1 Department of Environment and Resource Studies, Faculty of Integrated Development Studies, Wa Campus, University for Development Studies, Tamale, Ghana; 2 Department of Population and Health, College of Humanities and Legal Studies, University of Cape Coast, Cape Coast, Ghana; Sefako Makgatho Health Sciences University, SOUTH AFRICA

## Abstract

Mothers are recommended to exclusively breastfeed their infants for the first six months of their lives. Also, after the sixth month, breastfeeding should continue with added complementary foods to the diets of children. Studies designed to sought the views of mothers on breastfeeding practices are limited. The aim of this study was to explore challenges to breastfeeding practices by considering spatial, societal and maternal characteristics in Ghana. Twenty mothers aged 15–49 years were interviewed purposively in selected communities within two regions of the country. Thematic content analytical procedures were applied to interpret and present findings. Challenges (to both exclusive breastfeeding and complementary feeding) spanned across spatial (home and work places), societal, and maternal characteristics. Key themes identified were in relation to household chores, work schedules, family influence, low breast milk production, swollen breasts or sore nipples, access to food items and preparation or giving foods. Addressing these challenges would require co-creation of supportive environments between couples and significant others as well as tackling institutional barriers that obstruct adequate breastfeeding among mothers. On complementary feeding, there is the need to form community health volunteers help educate mothers more on how to appropriately use local foods to feed their children.

## Introduction

The importance of breastfeeding practices for a healthy growth and development of infants and young children have been presented in numerous policy documents [1–2]. Breastfeeding practices are categorized into two broad aspects; exclusive breastfeeding and complementary feeding [3]. For each of these aspects, guidelines are stipulated as to how to appropriately practice each of them to yield maximum outcomes for the good health of children. For instance, mothers are encouraged to exclusively breastfeed their infants for the first six months of their lives. It is worth mentioning that breastfeeding is much more than giving infants and young children breast milk. Breastfeeding is a complex adaptive process that bonds a mother and her child. During this process, physical, biochemical, hormonal, and psychological exchanges occur to facilitate the transfer of needed essential nutrients between the mother and her child [4]. After six months of exclusive breastfeeding, breastfeeding should still continue with added complementary foods as recommended by World Health Organization [5].

The introduction of complementary foods is necessary since after the sixth month, breast milk alone is no longer enough to meet the nutritional requirements of infants [5]. At this stage, it is a transitional period when infants are introduced gradually to family foods. Also, it is recommended that the feeding process should start with other milks and non-milk liquids; and as the children grow older, then other semi-solid or soft foods could be given to them [6]. Mothers in their quest to feed their children, as recommended, are sometimes confronted with both personal and external challenges [7].

Research indicates that mothers are sometimes negatively influenced by significant others as to how to practice breastfeeding [7]. Other challenges documented include the belief that breast milk alone is not sufficient in meeting nutritional needs of infants; short maternity leave period; and socio-cultural pressure to introduce water and artificial feeds [8–9]. Again, breastfeeding mothers who return to work often feel exhausted–since they feed-on-demand and attend to family and employment responsibilities–leading to concerns for their personal health [10]. Also, Nankumbi and Muliira [11] observed that challenges in relation to appropriate infant and young child-feeding practices are mothers' knowledge about complimentary feeding, influence of culture custodians on mothers, and patterns and burden of other responsibilities the mothers have in the household.

For mothers to practice appropriate complementary feeding, they need to have suitable and enough foods, and ensure that there is diversity in the diets they give to their children. However, in many resource poor contexts, foods that are available are predominantly cereal-based with low nutrient density and poor mineral bioavailability, posing substantial low nutrient consumption for breastfeeding mothers and their children [12]. Even in some environments, commercial fortified foods are often beyond the reach of financially-challenged-mothers; therefore, homemade complementary foods remain commonly used [13]. These homemade foods are mostly unfortified or unenriched plant-based complementary foods hence providing insufficient key micronutrients especially among children aged 6–23 months [14].

To promote infants and young children feeding issues, the Ministry of Health (in Ghana), has implemented a number of related policies including the Baby-Friendly Hospital Initiative, the National Child Health Policy and the National Nutrition Policy. The Baby-Friendly Hospital Initiative was adopted and implemented in qualified health facilities in the country to promote breastfeeding initiation and improvements of exclusive breastfeeding among neonates and infants less than six months respectively [15]. The National Child Policy was equally implemented to promote the survival, growth and development of all children [16]. Also, the National Nutrition Policy, as contained in one of its measures, was to promote optimal nutrition among infants and young children in the areas of appropriate breastfeeding and complementary feeding practices [17]. All these policies, as part of their purposes, were intended to guide stakeholders in the country to effectively and properly ensure the healthy growth and development of infants and young children. Notwithstanding the well-articulated aims of the of the aforementioned policies, infants and young children feeding practices are still low in Ghana [18].

Prior studies have documented the determinants, predictors, or risk factors of breastfeeding practices [19–21]; primarily skewed towards the positivist paradigm. This suggests that there is paucity of literature on exploring challenges mothers encounter in their quest to practice exclusive breastfeeding and complementary feeding. We, therefore, sought to explore views of mothers on challenges of breastfeeding practices in the areas of exclusive breastfeeding, and complementary feeding, drawing on an interpretivist approach. The study primarily focused on mother-child dyads. Findings from this study would highlight issues that impede the practice of these feeding practices among mother-child dyads. Salient views from the mothers might pin-point to how to improve infants and young children feeding practices in Ghana.

### Theoretical concepts of the study

This study hinges on Bourdieu’s *habitus* and *dispositions* concepts. These concepts are used to explains that “food and eating is much more than a process of bodily nourishment: it is an elaborate performance of gender, social class and identity” [22]. The concept *habitus* denotes social structures and history of individuals that interrelate to define perceptions and actions in relation to their social environment [23]. Also, the concept of *dispositions* depicts preferences. That is, the behavioural tendencies that a person exhibits due to cultural beliefs passed on to the next generation through unconscious memories of attitudes and practices [24].

Conceptually, the concepts of *habitus* and *dispositions* were used to understand challenges mothers encounter in relation to breastfeeding practices (exclusive breastfeeding and complementary feeding). Social structures may pose challenges for mothers in their attempt to comply with breastfeeding practices. The roles played by mothers at homes in the Ghanaian context are mostly stressful and sometimes time consuming [25]. In such situations, appropriately adopting exclusive breastfeeding and complementary feeding regimens may be hindered and hence influence their perceptions and actions. For instance, the type of the complementary food a mother gives to her child may be informed by how she perceives ‘food’; whether food is for the nourishment of her child or for satisfaction. How society defines food may challenge her intention of complementarily feeding her child. Again, the inability of a mother to provide adequate and appropriate foods for her child is partly determined by what a mother can afford and what foods society expects her to feed her child. Hence, the definition of food by a mother is being informed by social structures (*habitus*) and by preferences (*dispositions*).

## Methods

### Study setting

The study was conducted in two regions–Upper West and Western. We purposively selected these regions due to the level of exclusive breastfeeding prevalence for children age 0–23 months in each region following a secondary analysis of data from 2014 Ghana Demographic and Health Survey (GDHS). Comparatively, Upper West Region had the highest prevalence rate (33.08%) while Western Region had the lowest prevalence rate (9.60%) of exclusive breastfeeding. Convenience sampling was subsequently applied to select urban and rural settings within each of the regions. In Upper West Region, Kambali (urban) and Siriyiri (rural) were selected whiles Kojokrom (urban) and Inchaban Nkwanta (rural) were selected in Western Region.

Upper West Region is located in the north-western part of Ghana. It is predominantly inhibited by the *Dagaaba* and *Sissala* ethnic groups. It lies in the Guinea Savannah vegetation belt and it is a place commonly associated with the shea (*Vitellaria paradoxa*), baobab (*Adansonia*), dawadawa (*Parkia biglobosa*), and neem (*Azadirachta indica*) trees. Main economic activities in the region are agricultural related and key crops grown are maize, millet, and peanuts (groundnuts). Animal husbandry (for example sheep, goats, pigs, and cattle) is also common in the region. *Tuo-zafi* (a stable food made from maize or millet flour, eaten commonly in the northern part of Ghana),accompanied with soups made of green vegetable leaves is the commonest food among households in the region.

On the other hand, the Western Region is located in the south-western part of the country with the *Fante* being the largest group of inhabitants. About 75 per cent of the region is dense forest vegetation. The key economic activities include cocoa, rubber, oil palm and coffee farming. Food crops mostly cultivated in this region are plantain, cassava, and cocoyam. *Fufu* (pounded cassava with plantain, yam or cocoyam) with soup prepared from palm nut is a stable food in the region.

### Study design

An interpretive case study design was adopted to explore challenges of breastfeeding practices (exclusive breastfeeding and complementary feeding) among mothers in selected regions in Ghana. The application of interpretive case study design facilitated in discovering deeper and social meanings attached to breastfeeding practices. Data were collected through unstructured in-depth interviews. The interview guide was designed in English language. It was subsequently translated to *Fante* (for participants in the Western Region) and *Dagaare/Waale* (for participants in Upper West Region) and then translated back into English Language to ensure reliability of the data collection instrument. Interviews were conducted using any of the languages (English, *Fante*, *or Dagaare/Waale*) that a mother was conversant with, understood and spoke fluently.

The interview guide was structured in three main portions. The first portions contained questions on the background characteristics (age, education, marital status, occupation, age of child, sex of child) of participants. The second part had questions on challenges to exclusive breastfeeding, and the third part included questions on challenges to complementary feeding. These questions were in areas of individual, household, health, and food items. In order to get in-depth explanations and exhaust each of the issues under discussion, probes followed the questions in the second and third sections. The interview guide was developed based on a wider assessment of the data and questionnaire of the Ghana Demographic and Health Survey 2014.

### Participant selection and data collection

Criteria for the selection of mothers were that a mother should have had a singleton birth, be aged 15–49 years old and have an infant or a child of aged 0–23 months. Also, mothers had to be residents of the selected sites. The data collection tool was pretested in a rural setting community. Pretesting was done in two waves. The first wave consisted of one participant and the second wave involved two participants. This afforded the research team the opportunity to revise the tool for clarity.

The purposive sampling technique was used to select mothers. Interviews were conducted according to the date, time and place agreed upon with each mother. Some mothers allowed the interviewers to interview them only after we had introduced ourselves and requested for their consent (in either a signature or thumb print mark on the informed consent form). Those who were willing but could not afford the time for a prompt interview, a time and place were agreed upon. Interviews took place at either their homes or work places. In both instances, interviews were conducted without the presence of a third-party. This was done to avoid split attention on the side of mothers during the interviews and also to have clear audio recordings of the discussions. No initial sample size was set for the study. Twenty-two participants were contacted but two mothers in Western Region declined to be interviewed because they were busy and were not ready.

In all, 20 mothers (10 each from Upper West and Western regions) participated in the study. The other team members, who are experienced in qualitative studies tutored the interviewer (corresponding author) who then canvassed through the selected communities to recruit participants until data saturation was attained. This was attained when no new themes were emerging from the data collected [26]. All interviews were conducted between August 19, 2017 and September 17, 2017; and each interview lasted about 35–45 minutes. Prior to the data collection, no relationship was established with the participants. Informed consent was obtained from each participant and confidentiality of discussions was assured before the commencement of each interview. Unique numbers were then assigned to each participant to ensure anonymity.

Participants were informed of their rights to respond or not respond to any question they might deem sensitive and could pull out from the interview at any time. The interviews were audio recorded and field notes were written in a hand book. The field notes included issues such as date, time, exact location and phone number of mothers, and health status of mother-child pairs. Using the field notes and transcribed data from each interview, where necessary, interview questions were modified accordingly. The researchers obtained ethical clearance, with reference number UCCIRB/CHLS/2016/22, from the Institutional Review Board (IRB) of the University of Cape Coast, Ghana.

### Data analysis

Thematic analysis was applied using the inductive approach to analyze the data [27–28]. Preliminary data analysis started immediately with the first two interviews and transcription of data was done manually. Each transcript was critically read in order to become familiar with the data. These transcripts were verified by the research team with reference to the field notes where necessary. Some participants in both localities were subsequently contacted to clarify and validate issues in the transcribed data.

On the transcripts, identified emerged themes were marked with letters. For instance, the letter ‘A’ was used for exclusive breastfeeding challenges at home and ‘B’ for exclusive breastfeeding challenges at work place. Similarly, numerals were used to specifically identify sub-themes. For example, the number ‘1’ was used for household chores; and ‘2’ for formal and informal work schedules. Further, to enable easy identification of individual transcripts, unique codes (consisting of a number, place of residence, and region) were assigned to selected quotations.

Further, each researcher independently reviewed the transcripts and outlined themes. The research team met to discuss the identified themes. After the meeting, again, each researcher was tasked to review the initial themes that were put together by critically comparing them to the transcribed data. This iterative process was necessary to comprehensively identify further themes and categories that might have been omitted. Inconsistencies, where identified, were reconciled. Also, quotations used to support views expressed by mothers were mutually agreed upon by all researchers. Interpretations of views were done to reflect salient and subtle meanings as expressed by participants. Also, as the analysis of themes continued, each identified theme was interpreted coherently to show the meanings participants attached to challenges of breastfeeding practices noting their spatial and societal characteristics. The lead researcher wrote up the findings and the other team members, who are well vest in qualitative research, reviewed the presentation. Comments were presented accordingly and then a neutral qualitative researcher was called upon to peer review the presentation.

**Fig 1:**
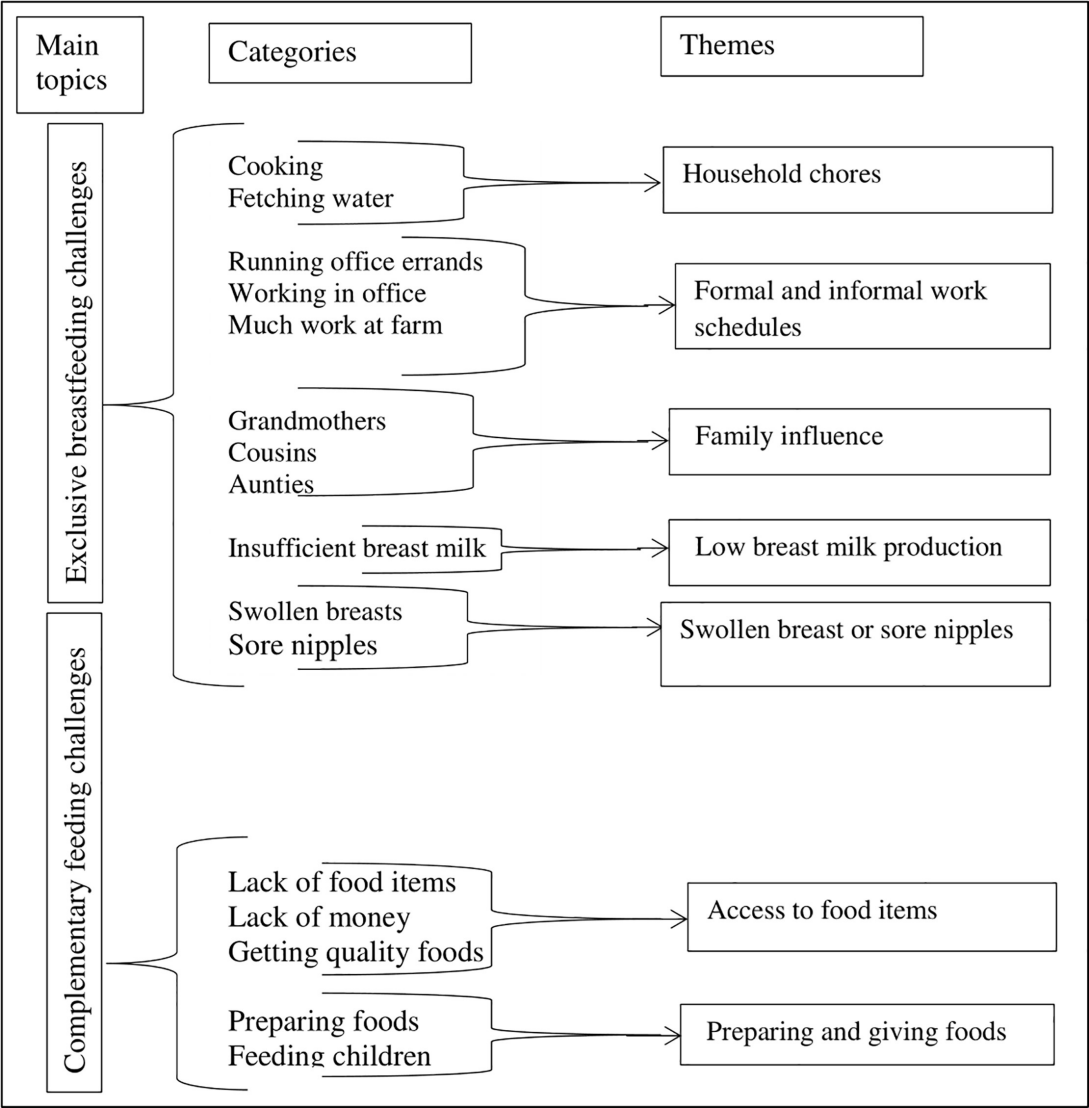
Description of coding tree showing themes. Exclusive breastfeeding challenges consisting of household chores, work schedules, family influence, low breast milk production, and swollen breast or sore nipples. Complementary feeding challenges consisting of access to food items, and preparing and giving foods.

In all, sixteen categories emerged from the challenges of exclusive breastfeeding, and five categories from challenges of complementary feeding (Fig 1). To ensure mutual exclusiveness of the categories, some categories were merged. This reduced the number of themes to seven. Due to the merger of categories, newly constructed typologies that were not indicated by the participants were used to label the themes where necessary. For instance, ‘formal and informal work schedules’ was used to represent both ‘running office errands’, ‘working in office’ and ‘farm works’. The themes identified were used as main headings in the results sections. The themes presented at the results section are: household chores; formal and informal work schedules; family influence on exclusive breastfeeding; low breast milk production; swollen breasts or sore nipples; access to complementary food items; and preparing and giving complementary foods to children.

## Results

### Background characteristics of participants

Results from the qualitative study indicated that 14 mothers were within the age group of 21–30 years (Table 1). Nine of them attained primary education. Eighteen mothers were married. Seven of the mothers were traders including one each as a teacher, a hair stylist, and a *Pito* brewer (a type of alcoholic beverage made from millet or sorghum in northern parts of Ghana, parts of Nigeria, and other parts of West Africa). An equal number of their children were within the age brackets of 6–11 months and 12–23 months. Female children were 12. There was an equal number of mothers from the regions (Western and Upper West), and place of residence (urban, and rural) within each region.

**Table 1:** Background characteristics of participants (N = 20).

Variable	Frequency
**Maternal age** *[mean = 26*.*85; SD = 5*.*274]*	
≤ 20	3
21–30	14
31–40	3
**Maternal education**	
No education	3
Primary	9
Junior High School	4
Senior High School	2
Higher	2
**Maternal marital status**	
Never married	1
Married	18
Ever married	1
**Occupation**	
Unemployed	6
Trader	7
Seamstress	2
Teacher	1
Hair stylist	2
*Pito* brewer	1
Weaver	1
**Age of child** *(months) [mean = 10*.*40; SD = 3*.*872]*	
0–5	2
6–11	9
12–23	9
**Sex of child**	
Male	8
Female	12
**Region**	
Western	10
Upper West	10
**Residence**	
Urban	10
Rural	10

### Themes

The key themes that emerged from interviews with the mothers were as follows: household chores; formal and informal work schedules; family influence on exclusive breastfeeding; low breast milk production; swollen breast or sore nipples; access to complementary food items; and preparing and giving complementary foods to children.

#### Household chores

It emerged from the study that cooking and fetching water were household chores that mostly interfered with the practice of exclusive breastfeeding. With mothers in Upper West Region, periods of cooking foods for their families were regularly mentioned. As indicated by these mothers, they cook their stable food *(tuo-zafi)* on daily basis and this sometimes pose challenges to the practice of exclusive breastfeeding in the home. A mother stated:

If you are cooking, especially *tuo-zafi* and the child is crying, you may find it very difficult to stop and breastfeed her. You would have to still leave her with someone until you finish. (A 34-year-old mother, Urban, Upper West)

Also, fetching water can be a stressful activity since some mothers have to distant walk to public service points to get water using gallons or head pans. Some of these sources of water, the mothers mentioned, are located at far locations and at times unsuitable to send their children along. Even when the need arises for them to carry their children along, they are mostly unable to breastfeed their children:

Fetching of water poses some challenges. You can’t carry water while the baby is breastfeeding. Most at times, you have to back the child until you pour the water. Sometimes too, you would have to leave the child at home and go to the borehole to fetch water. (A 24-year-old mother, Urban, Upper West)

#### Exclusive breastfeeding challenges in work schedules

The work schedules of mothers interfered with the practice of exclusive breastfeeding. Interviews with mothers revealed that handling children and carrying work schedules was not conducive since they cannot carry the child along when performing some errands. This sometimes affects how they exclusively breastfeed their children:

May be at work they asked you to go somewhere to do something, you may be forced to leave the child with the baby-sitter and go. You would not like to send the child wherever you are going. The time the child is supposed to breastfeed, on your return, that time would be past posing a challenge to the child. (A 30-year-old mother, Urban, Upper West)

Some mothers indicated emphatically that they do not have enough time at the work place. They are left with no option than to feed their children with other foods since the periods they may breastfeed them would not be enough while at work. A mother narrated:

Because I don’t have time in the work that I do so if I don’t give him food early and it’s only the breast milk that he is going to rely on, he will be hungry. As for breastfeeding, I breastfeed him but I have to add other foods too. (A 37-year-old mother, Urban, Western)

Again, others had such tight schedules set for them at their work places that they sometimes delay breastfeeding their children till they (mothers) are at home. This kind of tight schedules was cited by a mother as:

Assuming you created a portion in the farm and you must finish it before sunset so that tomorrow you continue at another place, and the baby is also crying. The only option would be to back the baby and continue working until you finish that portion. In the house, he can then suck. (A 38-year-old mother, Rural, Upper West)

#### Family influences on exclusive breastfeeding

Close associates such as grandmothers, co-tenants, and other relatives were found to be people who sometimes challenged mothers on the practice of exclusive breastfeeding. These individuals were widely mentioned in interviews with mothers in Upper West Region. Among them, grandmothers were commonly mentioned as posing challenges to mothers’ decisions on exclusive breastfeeding. Some grandmothers intentionally give substances such as water to children when bathing them. Their persistent suggestions and demands of giving water and foods to children who are supposed to be exclusively breastfed were worrisome for some mothers. A mother explicitly cited such incident:

When she [mother-in-law or grandmother] was bathing the baby, she used to fetch some of the bucket water into her [child] mouth. I told her [mother-in-law or grandmother] not to be doing that. She told me that, the nurses are doing that and killing people’s children. She said that, some children come to earth because of food and must be given the food. (A 30-year-old mother, Urban, Upper West)

Also, others, such as aunties and cousins were noted for giving foods to children (less than six months) without the notice of their mothers:

One day, I was cooking and gave the child to one of my aunties but she sent the child outside and gave her [child] ‘Pito’ [locally brewed alcoholic beverage]. I smelt the ‘Pito’ from her [child] mouth but my aunty denied giving her [child] such drink. Later, the alcohol was then working in her system and she cried heavily until my aunty then confessed that she actually gave her the drink. (A 19-year-old mother, Rural, Upper West)

#### Low breast milk production

Mothers reported that insufficient breast milk was a major challenge that hinder efforts towards attaining optimum exclusive breastfeeding especially among mothers in Western Region. Mothers who were unable to meet their children’s demands for breast milk found themselves in a state of despair:

Before she was one month, she used to cry a lot and most times I was not okay with it and when she breastfeeds too, it wasn’t enough for her so it became a problem. (A 26-year-old mother 5, Rural, Western)

In some instances, mothers who could not produce enough breast milk had no other choice than to give their children other foods to supplement the insufficient breast milk:

The child really likes crying and it was worrying me so I thought hunger could be a factor so I said let me try giving him food and see, because even when he has finished breastfeeding he would still be crying so that’s why I started giving him food. And I realized afterwards that it was indeed because of hunger that he was crying so I gave him food. (A 28-year-old mother, Urban, Western)

#### Swollen breasts or sore nipples

Mothers recounted moments when the health status of their breasts was a challenge for them to practice exclusive breastfeeding. A mother recounted how difficult it was to exclusively breastfeed her child due to her swollen breasts: “because I had a problem in one of my breast [swollen] … It was difficult to breastfeed” (Mother 3—Rural, Western). Another mother narrated how serious it could be to practice exclusive breastfeeding with boil or sore on the breasts: “assuming you have a boil or sore on your nipples it becomes a serious challenge to exclusively breastfeed”. (A 30-year-old mother, Urban, Upper West)

#### Access to complementary food items

The acquisition of food items by mothers to prepare the required complementary foods for their children was a challenge for most mothers in Upper West Region. The unavailability of preferred food items was a concern for some mothers. A mother described that it was really challenging for her to feed her child appropriately:

It was not easy. Somebody [child] who was only breastfeeding and later started eating porridge and other foods, it was not easy finding the food to sustain all of us as we the parents were not working. Everything we the parents eat, he would also eat the same. In fact it was not easy. (A 30-year-old mother, Urban, Upper West)

Other challenging moments were when mothers had insufficient funds to buy requisite ingredients to prepare foods for their children. In some of these moments, some mothers recount having portions of the items needed in preparing a meal but lacking other items due to lack of money to purchase the remaining items. A mother recounted:

The flour used to be available but the soup ingredients used not to be available due to lack of money. (A 19-year-old mother, Rural, Upper West)

Instances also existed when some mothers or families who have the financial means to purchase needed food items could not get them to buy. One mother sadly shared that:

At times getting the food items to purchase is a problem. You may not have the money to purchase them and sometimes too you may have the money but getting them to buy also poses a challenge. Getting quality ones to buy is not always easy. (A 34-year-old mother, Urban, Upper West)

The unavailability of foods, therefore, was a key issue mothers had to deal with in their quest to provide appropriate complementary foods for their children.

### Preparing and giving complementary foods to children

Interviews with mothers, particularly those working, further suggested that preparing complementary foods and feeding children appropriately was a key challenge. Also, mothers expressed concerns about the lack of means to keep the foods they have prepared warm:

Preparing the food is the problem. If there is no time to feed her at work and also when you send the food to the work place and it cools, you cannot feed the child as required. As you don’t have heater or microwaves or coal pot at work place, it becomes a serious headache. (A 30-year-old mother, Urban, Upper West)

Mothers indicated that feeding children adequately at work places was not easy. Most working mothers entrusted the care of their children to other people such as baby-sitters at work places. These people, the mothers indicated, might not have the motherly care to appropriately feed their children. A mother said:

The way the mother of the child would feed the child, the baby-sitter or any other person cannot feed the child like that. The other people would not have that time or patience to feed the child. So it is good that you the mother to always find that precious time to feed the child. (A 30-year-old mother, Urban, Upper West)

## Discussion

The interviews conducted provided insight into the views of breastfeeding mothers in relation to challenges they encounter in their bid to exclusively breastfeed as well as complementarily feed their children. This study identified nursing mothers’ engagement in performing household chores (especially cooking and fetching water) as challenges to practicing optimum exclusive breastfeeding. Nursing mothers being overwhelmed at combining exclusive breastfeeding and household chores can be attributed to weak social support systems. This in the light of increasing urbanization is leading to the rise of nuclear families compared to larger family systems (extended families) that use to offer needed social support for nursing mothers in Ghana [29]. The disintegration of larger families into smaller ones sometimes poses constraints that tend to hinder the practice of exclusive breastfeeding. In the Ghanaian context, extended families accommodate several relatives (aunties, cousins, nieces) who are mostly willing to offer assistance to nursing mothers. As communities get urbanized, supports from these relatives diminish or are unavailable. On the other hand, by tradition, husbands are not supposed to take part or assist in household chores since these activities are highly gendered towards females. Where necessary, some husbands offer minimal assistance in household chores that may not be enough to relief the burden on mothers. The absence or limited assistance nursing mothers get in performing household chores in most contemporary Ghanaian homes tend to negatively influence how their infants are exclusively breastfed including those who are complementarily fed.

Further, the findings revealed that at work schedules of mothers equally hinder the practice of exclusive breastfeeding. The early return to work by breastfeeding mothers in civil and public services—after three months of maternity leave—tend to compound exclusive breastfeeding challenges for mothers [30–32]. Aside the early return to work, most institutions or organizations in Ghana do not have breastfeeding cubicles or rooms as required by law. Again, the location of crèches and where most mothers work are distant. In such circumstances, mothers may be required to express breast milk to be given to children while they are at work [33]. Although this approach would be more useful elsewhere, the certainty that children might be given the expressed breast milk in a good condition in the context of Ghana is questionable. Likewise, in busy informal sectors such as trading and farming, mothers encounter challenges that impede the practice of exclusive breastfeeding. Mothers in their hustle and bustle at local trading centers would have to combine uncoordinated activities by attending to clients and at the same time trying to optimally breastfeed their children. As a result, exclusive breastfeeding is breached with early weaning inevitable [34–35]. Hence, mothers have no choice than to introduce complementary foods to their infants.

In addition, within societies, mothers may be challenged in their quest to practice exclusive breastfeeding. Grandmothers were commonly mentioned as individuals who have much influence on how mothers, particularly young mothers, should breastfeed. Particularly in Ghana, grandmothers (mother-in-laws) have much say in relation to health care of their grandchildren [36]. Grandmothers tend to act as advisors and sometimes authorize what kinds of foods to be given to children. The practices of giving infants herbs, water, and concoctions are common habits of grandmothers in some communities in Ghana [37] and elsewhere [38–39]. Nursing mothers (who are mostly daughter-in-laws) staying with the grandmothers, due to respect, may not be able to oppose to all actions grandmothers take even when that contravenes with the practice of exclusive breastfeeding. On the contrary, Aguree et al. [40] in their study observed that grandmothers with appropriated knowledge on the importance of breastfeeding rather tend to encourage mothers to practice exclusive breastfeeding. In addition to grandmothers, other family members sometimes introduce foods or liquids to infants who are supposed to be exclusively breastfeeding; such family members act without the knowledge of mothers. This finding collaborates with what Nduna et al. [41] found in Zimbabwe. They noted that even trusted individuals such as aunties who are supposed to act as co-mothers intentionally give liquid foods to children (less than six months) without the knowledge of mothers.

Again, in this study, low breast milk production was another challenge encountered by mothers. Low breast milk production is linked to medical and non-medical conditions [42]. Most often, it is the non-medical conditions (such as less sucking time, and delaying feeds) that contribute to low breast milk production among mothers [43]. For instance, less sucking time probably could be due to sore nipples resulting in low breast milk production. Also, while mothers may intend to exclusively breastfeed children, physical conditions may inhibit such intentions. For instance, breast abnormalities such as sore nipples [44] could lead to less breastfeeding and consequently low breast milk production. Mothers in this study pointed to all these as shortcomings that thwart their willingness to practice or continue with exclusive breastfeeding. Agunbiade and Ogunleye [45] documented similar findings in a study conducted in Nigeria. They identified constraints such as swollen breast, nipple problems, and perceived milk insufficiency among breastfeeding mothers.

Moreover, it was found that mothers had challenges accessing complementary food items or ingredients. Insufficient financial endowment poses challenges to some mothers in purchasing complementary foods. This is a common challenge for mothers in rural areas, as compared to their urban counterparts, where mothers are usually not economically endowed to afford children’s foods. Zahiruddin et al. [46] also found similar issues in their qualitative study which explored challenges and patterns of complementary feeding in India. Also, not having the requisite knowledge on where to acquire quality complementary foods for children was a related challenge for mothers. With the influx of so many low-quality complementary foods in markets, mothers need to be knowledgeable enough to buy appropriate foods for their children to ensure healthy growth and development [47]. Therefore, mothers not having enough money to buy food or without the required knowledge on the best choice of food, they may tend to feed their children to be satisfied with less regard to nutritional gains.

Preparing and giving complementary foods to children while at work was a challenge for mothers. Mothers are supposed to adhere to diversifying—adequate nutrient—rich foods and hygienic practices when preparing complementary foods for children [2]. Weaned children also need to eat frequently and consistently. This requires mothers to always prepare foods to ensure that their children are healthy. Aside this, foods prepared for children are supposed to be kept warm to avoid food contamination. Working mothers who may not have effective food storage containers to keep their children’s food warm are likely to give them cold foods. In instances where children are not fed because foods are cold, then the expected frequency and consistency in the feeding cycle of children would be breached. Children may even be exposed to consuming cold foods when other caregivers are to feed them in the absence of their mothers [46].

Findings of this study emanated from small sample of mothers within two regions, and views expressed by mothers are unique to their own observations and knowledge. The views may not represent those of other mothers in the same communities.

## Conclusion and implications

Findings suggest that mothers encountered exclusive breastfeeding challenges at home, with work schedules within their societies, and in relation to their health status. In order to address these challenges, we suggest the creation of awareness through especially radio for family members and other significant others to enable them provide adequate support for exclusive breastfeeding mothers. Also, increasing maternity leave to at least four months for expectant and nursing mothers by authorities in the country would reduce challenges nursing mothers have at work places. On complementary feeding, a platform should be created for midwives and community health nurses to provide further education on how to adequately and appropriately use local foods to feed their children. Besides, the provision of suitable feeding cubicles for breastfeeding or feeding children at work places by authorities would be a positive measure to ensuring mothers feed their children with ease.

## Supporting information

S1 File Data set. (RAR)

S1 Interview Guide (DOCX)
