# Supplementary material for: Maternal challenges of exclusive breastfeeding and complementary feeding in Ghana
Source: PLoS One. 2019 May 2;14(5):e0215285. doi: 10.1371/journal.pone.0215285 (PMC6497241; doi:10.1371/journal.pone.0215285)
Supplement: S1 Interview Guide — (DOCX) [file pone.0215285.s002.docx]

**INTERVIEW GUIDE**

**IN-DEPTH INTERVIEW TOPICS TO BE DISCUSSED WITH MOTHERS ON BREASTFEEDING PRACTICES**

**Introduction**

Thank you for agreeing to participate in the study. We shall have a short discussion to obtain your views on breastfeeding practices. We want to assure you that anything you say in this discussion will be confidential and that no identifying information will be recorded. Nothing that is discussed in this interview will be provided to anyone outside of the research. Do you have any question?

**This interview is meant for mothers with infants and children aged 0-23 months**

**(a) Introductory questions (warm up)**

- Number of children
- Plans to have more children

**(b) Background characteristics of participant**

| 1. Age of mother  2. Educational level attained  3. Marital status  4. Occupation | 5. Age of child  6. Sex of child  7. Region/Town |
| --- | --- |

**MAIN ISSUES TO DISCUSS WITH PARTICIPANT**

**1.0 Knowledge about breastfeeding practices**

**1.1 What is your understanding of exclusive breastfeeding?**

*Where did you get this information from?*

*When should a mother start exclusive breastfeeding?*

*What’s the expected duration of exclusive breastfeeding?*

*Why should a mother exclusively breastfeed her child?*

*Can exclusive breastfeeding prevent pregnancy? How?*

*What else does she know about exclusive breastfeeding?*

**1.2 What is your understanding of complementary breastfeeding?**

*When should a mother start complementary feeding?*

*How should a mother start complementary feeding?*

*What kind of foods should be given to a child as complementary foods?*

*What more do you know about complementary feeding?*

**2.0.** **Putting knowledge of breastfeeding practices into practice**

**2.1 How did you put or how are you putting exclusive breastfeeding into practice?**

*Immediately after delivery when did you start breastfeeding?*

*How long did you exclusively breastfeed your child?*

*Ask why she did or did not exclusive breastfeed for six months.*

*Do you mostly have sufficient breast milk?*

*Why are you exclusively breastfeeding your child?*

*Anything else on you exclusively breastfeeding your child?*

**2.2 How have you been feeding your child with other foods?**

*At which point in time did you give water to the child?*

*What kind of foods did you start giving to your child?*

*What are the compositions of each food given to your child?*

*How was each food given to your child prepared?*

*Do you add eggs, fish, groundnut pastes, milk and the like to your child’s food?*

*Why do you give each of these foods to your child?*

*Do you have anything else to say on the foods you give to your child?*

**3.0. Challenges of breastfeeding practices (exclusive and complementary)**

**3.1. What kind of challenges do you face at home when practicing exclusive breastfeeding?**

*What are your duties at home?*

*Do you have enough time to breastfeed?*

*Are your house chores too much to handle?*

*How does each of these duties limit you to exclusively breastfeeding your child?*

*Does your partner, other relatives or friends put pressure on you to give water to your child, or give some particular foods to your child?*

*What other challenges do you face in relation to exclusively breastfeeding your child at home?*

**3.2 With complementary feeding, what kind of challenges do you face at home?**

*Do you have enough time to prepare food for your child?*

*What are other things that make it difficult to appropriately feed your child at home?*

*Ask how each of the other things affect her feeding her child?*

*What are other challenges you face at home in terms of feeding your child?*

**3.3 At your work place, do you face challenges in relation to exclusive breastfeeding?**

*How do you work affect you exclusively breastfeeding your child?*

*Do you have enough time exclusively breastfeed your child?*

*Is there privacy to exclusively breastfeed your child at work? If no, why? If yes, what kind?*

*What else at your work place affects the way you exclusively breastfeed your child?*

**3.3 At your work place, do you face challenges in relation to complementary feeding?**

*What kinds of foods do you feed to your child at work place?*

*Why do you feed your child with each of these foods you mentioned at work?*

*Are these foods mostly warm or cold?*

*Why do you feed your child with warm or cold foods at work place?*

*Is there enough time to feed your child with other foods at your work place?*

*What else affects the way you feed your child at work place?*

*How do each of these things affect you feeding your child?*

**3.4 Do you have other challenges?**

*On breastfeeding (sored nipples, insufficient breast milk, child’s refusal to breastfeed, traditional beliefs, etc.).*

*On complementary feeding with other foods (insufficient protein foods [fish, milk], traditional beliefs, insufficient foods, etc.)*

**4.0 Closing key comments**

*Is there anything more you would like to add?*

**Thank you**
